# The Sovereign Ascendant: Financial Collapse, Status Anxiety, and the Rebirth of the Sovereign Citizen Movement

**DOI:** 10.3389/fsoc.2019.00076

**Published:** 2019-11-26

**Authors:** Edwin Hodge

**Affiliations:** Department of Sociology, University of Victoria, Victoria, BC, Canada

**Keywords:** Sovereign Citizen, radical citizenship, extremism, anti-government, social movements

## Abstract

As many scholars have noted, periods of economic or social unrest often bring about the growth or resurgence of extremist social movements on both the political left and the right. The 1990s saw the rise of the American militia movement, largely in response to the emergence of international organizations like the North American Free Trade Agreement, the General Agreement on Tariffs and Trade, and an increasingly internationalist American administration. Earlier still, during the agricultural depression of the 1920s, the chaotic social, political, and economic order proved to be fertile ground for the resurgent Ku Klux Klan. In the years immediately following the global financial collapse of 2008, the United States—and to a lesser extent Canada—saw the resurgence of the Sovereign Citizen movement, a puzzling, conspiratorial social, and political philosophy that sought to emancipate its adherents from the tyranny of an oppressive, dictatorial, and increasingly unstable system of corrupt and illegitimate states. This paper examines the origins of the Sovereign Citizen movement and illustrates the ways in which the movement's members deploy a radical concept of citizenship, rooted in conspiratorial thinking and often in direct conflict with the state to help manage status anxiety and uncertainty.

## Introduction

“*UCC 1-207, without prejudice I, Am Secured Party Creditor Charles Robert Barefoot*© *T.O.C. Pro-Fer, Pro-Se Natural Person (MAN) B.B.C. Sovereign American Citizen Court Appointed Self-Representation of Counsel Attorney-Of-LAW*^1^.”

Anti-government sentiment in the United States and Canada is nothing new. Indeed, an argument could be made that the revolutionary attitudes that underpinned both American and Canadian statehood were expressions of anti-government sentiment. In the late twentieth and early twenty-first centuries however, there has emerged a newer, more radical form of anti-government ideology in the form of the so-called “sovereign citizens” movement. Distrustful of state authority, often paranoid, and prone to extreme patterns of conspiratorial thinking (Douglas et al., 2017),^1^ Sovereign Citizens represent a growing challenge to law enforcement, municipal authorities, and the criminal justice system not only in the United States, but increasingly in Canada. Sovereign citizens (or “freemen on the land” as they are often called in Canada) grew from the Posse Comitatus and anti-tax movements in the American Midwest in the 1970s and 1980s, but in the years following the global financial crisis of 2008, the movement has found new life and is now enjoying a new popularity and reach.

Using this movement as a case study, this paper explores one pathway through which radicalized groups or individuals can make use of counter-memory and counter-memorial to construct alternative narratives of citizenship. Sovereign Citizens activities and protests are rooted in a belief that by enacting specific patterns of speech and behavior, an individual may formally withdraw their consent to be governed by traditional state authority. By engaging in a new, radically individualistic form of “protest-citizenship,” Sovereign Citizens challenge contemporary understandings of what it means to be a citizen of the neoliberal state.

This paper traces the origins of the Sovereign Citizen movement in the United States and Canada and illustrates how the radical, anti-government ideology's extreme individualism allows it to be easily adapted by non-American followers. Additionally, this paper explores the radical redefinition of citizen used by the movement and illustrates how the withdrawal of both a recognition of the authority of the state and the withdrawal of consent to be governed represents an attempt on the part of Sovereign Citizens to manage status anxieties rooted in fears of an increasingly globalized and interconnected world. This paper argues that the global financial collapse of 2008 fueled a significant growth in SovCit activism, which provided direction, meaning, and a strategy for anxiety management in the face of social, political, and economic change.

## Sovereign Citizens: Paper terrorism and a history of violence

The Sovereign Citizen movement can trace its ideological roots back to the early Posse Comitatus and anti-tax movements of the 1970s, 1980s, and 1990s, when groups of primarily white men organized around the principle that the highest law of the land was that of the county, specifically the office of county sheriff (Chaloupka, 1996). In these early iterations, anti-government groups sought to challenge the power of the federal government, which they viewed as corrupt and tyrannical, by empowering local groups of armed citizens to enact the law of the land within their respective areas. Part of this call to action was a declaration that the federal government was illegally extracting taxes from county citizens in violation of their natural rights During the height of militia, patriot, and anti-government activism in the United States in the 1990s, the primary aim of the Posse Comitatus movement was to minimize the influence of the federal government in the affairs of individual citizens—especially when it came to issues of taxation and land-ownership/use (Federal Bureau of Investigation, 2002).

Like the earlier Sagebrush Rebellion in which people in the American mid-west demanded greater state and county access to federal lands, the Posse Comitatus movement rejected the supremacy of the federal government's control of federal land and declared that Washington's role in land-management should be as minimal as possible (Sullivan, 1999). To bolster their position, Posse Comitatus members would often arrive at town-hall meetings or state committee meetings bearing weapons and declaring their intentions to protect their land and private property from government incursions (Chaloupka, 1996). These same arguments would resurface in twenty-first century SovCit rhetoric, as was seen during the so-called “occupation” of the Malheur National Wildlife Refuge by Ammon Bundy (Fantz, 2016), a rancher and anti-government activist associated with the Sovereign Citizen movement.

In the first half of the 1990s, groups like the Posse Comitatus tended to be clustered by observers and researchers under the rubric of “tax protest” (Dollar, 2013) movements, but in the aftermath of the 1995 Oklahoma City bombing, the activities of the movement caused it to be categorized as a Far-Right extremist group, akin to the white supremacist and militia groups whose numbers had been steadily growing throughout that decade. While Posse members were quick—and loud—in their protests at being defined in such a way (Larizza, 1995; Chaloupka, 1996), there was enough cross-pollination between the various movements to warrant that conclusion. In some cases, militia members would speak at meetings using rhetoric pulled from the literature of the Posse Comitatus; in other cases, members of racist organizations like the Christian Identity movement would draw on the anti-tax, anti-government rhetoric of the Posse during their speeches and sermons (George and Wilcox, 1996). Some researchers have argued that what some see as cross-pollination, others might rightfully interpret as “conflation”; in the media frenzy following the Oklahoma City bombing, law enforcement agencies, news organizations, and activist groups were quick to establish ties—however tenuous—between the various groups of the political Far Right (George and Wilcox, 1996). Certainly it is true that few formal ties existed between many of these groups—there were few “card-carrying” militiamen who were also “card-carrying” sovereign citizens—but arguments grounded in issues of membership overlook the glaring ideological similarities among these same groups.

As the Posse Comitatus movement grew, it evolved; new pseudo-legal bodies formed within Posse groups which claimed to speak with the authority of “natural” or “common” law and which movement members claimed superseded the legal system already in place (Chamberlain and Haider-Markel, 2005). These new bodies—called “Common Law Courts” by movement adherents—enabled individuals to dispense judgments on people or organizations that were claimed to have committed crimes against the movement or its members (Pitcavage, 1997). The most common tactic in the arsenal of these “Common Law” courts was to issue false liens against the homes and businesses of their enemies which were deleterious to the victim and costly to remove. In most cases, these liens took the form of statutory, common-law liens designed to damage a victim's credit rating or make it difficult—if not impossible—to sell a home or property (Chamberlain and Haider-Markel, 2005). By the end of the 1990s, the use of such liens—dubbed “paper terrorism” by government sources—had become a weapon in the arsenals of Posse groups and the militias alike (Loeser, 2015). During this same period, the use of the term “Posse Comitatus” to describe the constellation of anti-tax beliefs and groups had been supplanted by the term “Sovereign Citizen,” mirroring a change in the language used by adherents to reflect a renewed focus on personal liberty secured through absolute ownership of personal property—including land.

The 1990s and early 2000s also saw the growth of a new kind of rhetoric within the movement, which revolved around conspiratorial claims about the “true” nature of the United States government. While groups on the Far-Right have had a long history of conspiratorial thinking, a great deal of the newer rhetoric was both conspiratorial and apocryphal in nature. In the early 1980s, Gordon Kahl, a Posse Comitatus member, alleged that the United States government was secretly controlled by Jewish conspirators and was therefore not only illegitimate, but actively hostile to the interests of Americans like him (Berger, 2016). After Kahl was killed in a shootout with federal agents after killing two U.S. Marshalls in 1983, he gained martyr status within the movement, and his views were more widely disseminated. After the government crack-downs on patriot and militia groups in the years following the events at Waco, Texas, and the tragedy of the Oklahoma City bombing, Far-Right rhetoric became increasingly radicalized, often involving visions of open war against the American government, in addition to the racist and anti-Semitic elements espoused by men like Kahl (Churchill, 2010). This turn toward the apocryphal also coincided with the rise of global online communications and within the context of a rapidly globalizing world that saw the relocation of large numbers of manufacturing and working class jobs to overseas territories (Gallaher, 2000). As the movement evolved throughout the first decade of the twenty-first century its rhetoric became increasingly conspiratorial; the American government was no longer merely corrupt, it was also controlled by foreign—often Jewish—banking interests or cabals of shadowy forces intent on creating a New World Order under a single world government. What had been a rather fringe set of beliefs espoused by men like Gordon Kahl had become core elements of online SovCit discourse. By this time a new strand of belief had entered the collective lexicon of the “Sovereign Citizens” movement: Redemptionism.

### Emancipation Through Redemptionism

“*My straw man is an artificial person created by law at my birth on September 1, 1948 via the inscription of an ALL-CAPITAL LETTERS NAME on my birth certificate/document, which is a document of title and a negotiable instrument. My lawful, Christian name of birthright was replaced with a legal, corporate name of deceit and fraud. I, Thomas-Joseph: Kennedy have been answering when the legal person, KENNEDY, THOMAS JOSEPH, is addressed, and therefore the two have been recognized as being one and the same. When, I, Thomas-Joseph: Kennedy, the lawful being distinguish myself as another party than the legal person, the two will be separated* (Kennedy, 2016).”

In Sovereign Citizen parlance, “redemption theory” is the process by which a person can separate themselves from their “straw man.” In the ideology of the movement, a “straw man” is a fake identity used by national governments as collateral against foreign debt. This “straw man” is represented on legal papers through the capitalization of the name and by placing the surname before the given name. As the quote above indicates, whenever this form of a person's legal name is used, anything (like a legal document, charge, or fine) attached to it is applicable only to the “straw man” and not to the “lawful being.”

In the case of Mr. Thomas-Joseph: Kennedy, his birth certificate was sold, he claims, to the “Canadian Ministry of Industry Trade and Commerce” (Kennedy, 2016) at the time of his birth and used as collateral to obtain a loan from the Bank of Canada, which it then invested in stocks and bonds. According to this, Kennedy and anyone else who can emancipate themselves from their “straw man,” can thereby gain control over the money attached to it; they are also free from the control of the state that, according to redemptionist arguments, is only capable of controlling the actions of “straw men” and not sovereign persons like Mr. Kennedy (Berger, 2016). The manner through which the flesh-and-blood person gains control over their “straw man” is arcane to say the least, rooted as it is in Sovereign Citizens' use of pseudo-legal jargon and magical thinking (Dagnall et al., 2015) but in practice, the belief is that the Sovereign Citizen can issue bills or “IOUs” against the value of the “straw man” that are the equivalent of state-backed currencies.

When reasoning like this inevitably fails to deliver on its promise of economic and political freedom, Sovereign Citizens often resort of threats of violence against lawmakers, judges, and law enforcement agents, and sometimes engage in overt physical violence as well. In April 2008, while in jail awaiting trial on charges of tax evasion and fraud, Sovereign Citizen Robert Beale used the jail's phone to convene a “common-law jury” to threaten a U.S. District judge, Ann Montgomery. In addition to uttering threats against the judge, Beale also issued property liens and even issued an arrest warrant for Montgomery saying “God wants me to destroy the judge… That judge is evil. He wants me to get rid of her… Once I take down Ann Montgomery, no judge in the whole court will have anything to do with me (Sanchez, 2009).”

Beale is hardly alone. In 2001, former Klan leader and Sovereign Citizen Charles Barefoot was charged with conspiring to blow up the Sampson County sheriff's office in North Carolina. During the trial, Barefoot made heavy use of Sovereign Citizen defense tactics, which resulted in the judge declaring him not competent to stand trial because he “… does not possess a rational understanding of the proceedings against him (Southern Poverty Law Center, 2016).” While such rulings do occur, research into the psychologies of SovCit adherents has argued that many are competent to stand trial, despite the bizarre configuration of their conspiratorial beliefs (Pytyck and Chaimowitz, 2013).

### Cross-Border Growth

The Sovereign Citizen movement and related American protest movements have a long history of cross-border adoption particularly in Canada, where Canadian anti-government and anti-tax protestors have made heavy use of the ideology and adapted it to the legal landscape of Canada—with mixed success. In the case of Mr. Kennedy, his redemptionist strategy in dealing with the Canadian government included a heavy reliance on the “UCC” or “Uniform Commercial Code” (Matheson, 2018), one of many proposed uniform acts designed to harmonize interstate commerce within the United States and which has no force or effect in the Canadian legal system. Unsurprisingly, Mr. Kennedy reported that none of the government agents he spoke with were familiar with the UCC and informed him that American laws had no weight in the Canadian legal system. Mr. Kennedy took these statements as proof that his demands were legitimate. In his writings, Mr. Kennedy indicated that he viewed the Canadian government's refusal to accept his claims as evidence that not only was the Canadian government aware of its obligations under this piece of American legislation, but that it was afraid of Mr. Kennedy's knowledge of this “fact” (Kennedy, 2016).

Through online communications, SovCit ideology has spread quickly. Across YouTube and online forums examples abound that show remarkable similarities between the rhetoric of Canadian Sovereign Citizens (or “freemen on the land”), and their American counterparts, including the use of “common law courts,” the emphasis on specific spelling and grammar to distinguish “flesh-and-blood persons” from their corporate “straw men” (Wakefield, 2019), reliance on the Uniform Commercial Code and a habit of using “truth language” in their dealings with government officials. This “secret” legal language consists of terms designed to force government agents and organizations to acknowledge the sovereign power of the flesh-and-blood person over their corporate “straw man^2^.” The belief in the existence of a “secret” legal language that can compel courts and governments to capitulate to the will of the Sovereign Citizen is a form of magical thinking, a phenomenon with a long history in conspiratorial circles and one which some observers have argued sits at the root of many strands of anti-government and anti-tax ideologies (Pyke, 2016). Despite more than three decades of negative exposure, including stern refutations of the core beliefs of the movement by media and legal agencies alike, the Sovereign Citizen movement continues to grow and spread across North America.

According to a bulletin released by the Law Society of British Columbia in 2012, there are ~30,000 Sovereign Citizens active in Canada which represents a significant growth of the movement outside of the United States (British Columbia Law Society, 2012). These Canadian Sovereign Citizens engage in similar acts of protest or fraud as their American counterparts, often employing nearly identical tactics despite the different legal environments in which they operate. In one case a Sovereign Citizen approached a British Columbia notary and demanded that they notarize a “True Bill and Notice of Accounting” for $3.5 billion dollars, to be levied against the BC court of appeals. The notary witnessed the document and was later disciplined by the Society of Notaries Public for violating their rules (British Columbia Law Society, 2012). In response to an increase in these sorts of encounters, the Law Society of BC issued advisories to law firms throughout the province to prepare emergency security plans to ensure the safety of their employees. In another significant case, Alberta Associate Chief Justice Rooke in his decision in *Meads v. Meads*, compiled an extensive dissertation on the tactics, beliefs, and legal practices of the Canadian SovCit movement where he identified them as a species of vexatious litigant, and spent considerable time and energy compiling extensive notes on the movement (Meads v. Meads, 2012).

Reactionary movements like the Sovereign Citizens movement are often cyclical in nature, going through periods of resurgence and abeyance (Sanchez, 2009), yet for the SovCit ideology, the trigger is often sharp economic downturns or instabilities that threaten people with foreclosures or the erasure of their lifesavings (Sanchez, 2009).

The cross-border adoption of what was historically an American-focused anti-tax and anti-government protest is an example of the radical individualization of the concept of citizenship, a move away from notions of collective identity and association with traditional nation-states. Historically, citizenship has been understood as membership in a community that is tied to the modern nation-state (Brodie, 2002; Bosniak, 2003; Fudge, 2005), but for many in the Sovereign Citizenship movement, the nation-state is not only illegitimate, but also incapable of granting “true” citizenship to anyone. Citizenship flows from property rights and natural law, and while some Sovereign Citizens declare their citizenship to the county or to the state or province, many others see their citizenship as extending no further than their family or even beyond themselves.

## The radical citizen: individual, conspiratorial, and counter-memorial

### Citizenship Theory

While definitions of citizenship vary depending on which literature is being consulted, there is a general agreement that citizenship is associated with certain key considerations: community membership or inclusion, self-governance and the guarantee of entitlements or rights (Fudge, 2005). There is also literature that argues that citizenship is perhaps more correctly thought of as a process through which states and societies determine who is to be excluded from access to rights or entitlements (Lister, 2000). In both cases, however, citizenship has traditionally been attached to the nation-state; people were citizens of a specific state and granted access to rights or entitlements only within the confines of the state to which they belonged.

A sociological investigation of citizenship however must take on a different form, one that is primarily interactionist in orientation (Glen, 2010). While it is generally the case that citizenship includes as core components formal documentation including birth certificates, social insurance numbers or social security numbers and passports, citizenship also entails interaction between state and local actors, between community members and between communities and outsiders. As T.H. Marshall famously pointed out in his post-World War II examination of social life in Britain, there exists an important distinction between what he termed *formal citizenship* and *substantive citizenship* (Marshall, 1964). Under this articulation, formal citizenship consists of those components of citizenship that involve formal recognitions of rights, entitlements, and membership of individuals within a community or polity, while substantive citizenship can be thought of as the day to day *processes* of citizenship whereby individuals enact the patterns of behavior that give meaning to the formal recognition of citizenship by the state. Citizens can therefore be both formally recognized as citizens while at the same time prevented from participating in the affairs of their communities or state through informal barriers like race-based segregation or gender-based social inequalities.

The distinction between formal and substantive forms of citizenship is made even more apparent as states integrate into a globalized neoliberal economy; formal recognition of citizenship retains its distinctive focus on recognition through documentation—though increasingly the focus has been on working visas, student visas and passports as citizens frequently engage in cross-border and international systems of labor (Adelman, 2002; Andreas, 2003)—while at the level of day-to-day practice, citizenship has come to include routine cross-border exchange and informal systems of income generation.

Despite the popularity of Marshall's theory of citizenship—including his development of the three elements or types of citizenship (civil, political, and social)—it remains a quintessentially macrosociological model which downplays the interactionist component of social life (Colomy and Brown, 1996). In each of Marshall's categories, there remains a presumption of a uniform distribution of rights to all recognized members. Citizens were granted rights to thought or speech, or rights to personal property (civil), rights to participate in electoral politics as either an elector or elected official (political), and the ability to live in a society with access to social welfare and services such as public education or pensions (political). Therefore, formal recognition of citizenship rights is associated with the freedom to travel, speak one's mind, hold beliefs free from censorship or government intrusion, and similarly guaranteed rights but when examined microsociologically and interactionally, citizenship becomes a site of struggle, resistance and marginalization whereby the rights and entitlements granted by the state are infused with questions of race, gender, class, and creed. At this level of analysis, processes of citizenship are studies in differential access; citizenship is enacted unequally because citizenship as a concept is unequally granted. The microsociological level of analysis is also the most appropriate one for examining the “radical citizenship” of the Sovereign Citizen movement, as it is precisely the authority—and even the existence—of state-level citizenship that Sovereign Citizens reject.

### The Radical Citizen Constructed

The pathway traveled by Sovereign Citizens on their journey toward liberation is a convoluted one. Sovereigns must first commit to delegitimizing the institutions of the nation-state in which they reside, through the construction of counter-memory that rewrites history in conspiratorial tones (Burlein, 1999). “Counter-memory” can be defined as “… a complex mix of narrative, displacement, shared testimony, popular culture, rumor, fantasy, and collective desire… entangled with cultural products and imbued with cultural meaning” (Sturkin, 1999). Counter-memory is a subaltern narrative constructed by culturally marginalized or isolated groups that serves to bind them together in common practice or outlook. In the case of social protest, counter-memory—or counter-memorials—can be deployed as a way of resisting hegemonic cultural narratives (Bold et al., 2002). In the hands of activists working toward social justice, counter-memory, and counter-memorializing become powerful tools through which to resist erasure (Spaulding, 2014); in the hands of counter-cultural movements like white supremacists, patriot groups or the Sovereign Citizens, counter-memory becomes a tool for re-writing history to delegitimize state-level institutions or demonize ideological enemies (Hodge, 2011).

Sovereign Citizen counter-memory allows Sovereign Citizens to claim oppression at the hands of the state through taxation, “burdensome” regulations like driver's licenses, insurance or property taxes, or through the “illegitimate” use of force or threats of force by law enforcement agents in the execution of their duties. In some cases, adherents of SovCit ideology refuse to comply with law enforcement agents if they deem the agents' actions violate “natural law” or their rights as “free human beings to travel across Mother Earth” (A1 Talisman, 2018). In SovCit ideology, national governments are delegitimized through strategic deployment of counter-memory; contemporary nation-states are viewed as little more than corrupt shells of their former selves, controlled by foreign financial or corporate interests. Nation-states are further delegitimized in Sovereign Citizen circles through their participation in international institutions like the United Nations, International Monetary Fund, World Bank, or free trade systems like NAFTA. In each case, Sovereign Citizens argue that the sovereignty and therefore legitimacy of the state has been eroded through adherence to—or even acknowledgment of—international treaties and conventions, to the point where the state is no longer able to effectively guarantee property rights of its citizens (Southern Poverty Law Center, 2016). What makes this strategy so appealing is that it empowers SovCits and other radicalized actors (Torok, 2013) to see themselves as truth-seekers, who recognize that the hegemonic memorialization that reinforces and re-inscribes state power as natural is a mirage designed to establish a particular set of power-relations—that of the state over the individual. By this framing, the memory-work (Spaulding, 2014) of the state enables it to encourage citizens to forget; to forget they were ever free; to forget that state power is supposed to be a manifestation of the will of the People.

A common example cited by many Sovereign Citizens is the existence of the United Nations Conference on Environment and Development's Agenda 21, an expansive multinational agreement recognizing the importance of working toward a sustainable model of economic growth which emphasizes the need to consider the environmental impacts of development (United Nations Environment Programme, 1992). In the eyes of Sovereign Citizens however, Agenda 21 represents abandonment of national sovereignty and the beginnings of a one-world government ruled by a totalitarian regime (Harman, 2015). As recently as March, 2019, conspiracist websites like Beforeitsnews.com continue to “sound the alarm” about Agenda 21, declaring that the program will control what Americans can drive, what they can eat, what jobs they can have, and will destroy “the very concept of our Constitutional Republic” (DeWeese, 2019). For many Sovereign Citizens, the problem is clear: the tyrannical power of the state has encouraged its citizens to forget their sovereign rights and powers, and to allow themselves to be subordinated beneath an undemocratic international order they are unable to resist.

In the absence of a legitimate governing authority, Sovereign Citizens manifest their discursive counter-memory through counter-memorialization^3^ often through the self-issuing of documentation that had previously been issued by the now-illegitimate national government. Every cardboard license plate (Lenz and Potok, 2015), every home-made passport, flag or driver's license is an act of counter-memorializing a Sovereign Citizen's withdrawal of consent to be governed by institutions they have deemed corrupt beyond redemption.

Through counter-memory and counter-memorial, Sovereign Citizens undo the facts of the world they wish to separate from and engage in their own resistance memory-work. In their counter-memorials, a Sovereign Citizen is a person set apart from their neighbors and with that separation comes the freedom to act to reclaim what many sovereigns see as the ultimate prize: perfect and unfettered property rights, derived from natural or common law and free from state intrusion or regulation. The Sovereign Citizen must act to engage the state through radical citizenship; the Sovereign Citizen on equal footing with the institutions of the state. In their confrontations with the state, the Sovereign Citizen first repudiates the formal citizenship offered through state apparatuses, then further repudiates the *substantive* citizenship articulated by Marshall by refusing to acknowledge both the formally recognized citizenship of other members of their society and by denying that any legitimate informal or substantive citizenship practices can take place between them and their neighbors.

Sovereign Citizen counter-memory reduces non-sovereigns to the status of sheep or drones and therefore incapable of deliberate interactions as citizens. Under both Marshall's framework of substantive citizenship and later interactionist frameworks, authentic citizenship emerges from sustained interactions with other members of the community: law enforcement agents, public health and education representatives, business owners as well as private citizens but for many of these social actors, their dealings with Sovereign Citizens will be combative, aggressive, and tense as SovCits refuse to acknowledge their positions or view them as extensions of a tyrannical authority (and thus as illegitimate by definition). For SovCit adherents, the reconstruction of citizenship through the vocal rejection of implicit membership in social contracts is predicated on counter-memory.

The work of constructing the radical citizenship of the Sovereign Citizen movement is one of stages. First, the SovCit uses counter-memory and counter-memorial to delegitimize hegemonic narratives of state authority and nation-state-based citizenship. The SovCit then uses their new discursive reality as a foundation for working through the next stage whereby they begin the process of disentangling themselves—their true, flesh-and-blood selves—from the corporate shell through which the state has controlled them. Once “free” of the encumbrances—and debts—of their corporate “straw man,” the now-Sovereign Citizen can reclaim their “natural” property rights, and to claim the money they feel they are owed by the state for the unlawful use and sale of their corporate shells to foreign interests. This is accomplished through “truth language” and direct action against state agents; if the SovCit needs to pay a fine, they can “print” their own currency, if they need to travel, they can draw up their own passports, licenses or vehicle registration. Any debts they have incurred to the state are nullified by fiat; any money they owe to businesses or through rental agreements can be paid using personal currency. They are no longer bound by notions of nation-state citizenship; many SovCits no longer see themselves as even citizens of a community. They are sovereign in-themselves and free; they are immensely wealthy due to their new control over their corporate “straw man.” The state, should they choose to acknowledge its existence is no longer something to which they are beholden, as membership in the state is only ever done voluntarily (Levin, 2001). They manifest an exceptionally literalist interpretation of Lockean principles whereby in the instant that a government can no longer guarantee the property rights of its subjects, it ceases to be legitimate (Levin, 2001).

At its core, the Sovereign Citizen movement is about the (re)acquisition of power by those who feel powerless. The movement convinces them that the frustration they feel at being subject to state authority and administration and the vulnerability they experience in the shifting economic fortunes of an increasingly globalized economy are in fact evidence of oppression and the impetus to change. This configuration of practice can be seen in the reaction to the global financial collapse of 2008, when hundreds of thousands of Americans and Canadians, in the face of economic catastrophe, redefined themselves and their relationship with the state in the hopes that by doing so, they could regain the power they felt they had lost.

## The sovereign ascendant: the 2008 collapse and the rise of the radical citizen

### The Radical Citizen Deployed

For many North Americans, the global financial collapse of 2008 justified the apocalyptic rhetoric of late 1990s conspiracy theorists, many of whom turned to fringe beliefs as a way of establishing control over their chaotic circumstances (Douglas et al., 2017). In the span of a few short months, millions of Americans were left without work, without savings, and many without homes. The global crisis vaporized life savings and for many, heralded a new chapter in the struggle to find precarious employment in an economy already on its knees. For many Sovereign Citizens and members of related patriot groups however, the financial collapse wasn't simply a tragic series of events, it was also intentionally fabricated at the hands of Democrats in the United States government (Alexander, 2008). Others on the ideological fringes of the Far Right declared the collapse to be the work of Jewish influence in international finance, or through direct control of American presidents (Smith, 2016). Among Sovereign Citizen and patriot groups, the anxieties that emerged as a result of the financial collapse were compounded by fresh fears that an UN-backed, secret military operation was underway in the United States where foreign troops were being ferried into the country and hidden away in Federal Emergency Management Agency (FEMA) camps and nature preserves, while the government was at the same time preparing to go door-to-door to confiscate Americans' guns (Zaitchik, 2010).

The anti-government position of Sovereign Citizens, and indeed the reconstruction of the very notion of citizenship as local, grassroots, and rooted in explicit consent reflects a deep suspicion of the relationship between state and international systems of governance. It is common to find among Sovereign Citizens parallel beliefs in the existence of super-national conspiracies to eliminate national sovereignty or impose “globalist-driven” hierarchies of power or control. Not only does this manifest in anti-globalization, anti-United Nations conspiracies, but also in dog-whistled anti-Semitism (“Globalist” becomes a stand-in for “Jewish” influence in national politics) (Powers, 2019).

By 2011, the number of extremist groups in the United States—including Sovereign Citizen groups—had spiked from 149 in early 2008 to more than 1,274, a rise which coincided with the worst years of the global financial crisis (Johnson, 2012). A part of this sharp rise can be explained by examining the core beliefs of the movement itself, which views the loss of property rights to be a grave offense to the natural rights of citizens; the wave of foreclosures that occurred during the financial crisis would be the very height of government excess and abuse by anyone sympathetic to the Sovereign Citizen viewpoint. In such an environment, desperate people, under threat of losing their homes and properties, turned to the emancipatory rhetoric of Sovereign Citizens to help them keep their properties and savings. In the depths of the global financial crisis, the radical citizenship of the Sovereign Citizen could be activated and deployed to help adherents manage threats to their property and social status; if the government is illegitimate and acting tyrannically, then nothing it does to the SovCit's property is legal, and by becoming aware of this, the SovCit becomes free from it.

By the early 2010s, Sovereign Citizens had begun challenging the legal systems across North America by issuing false liens against public properties, or by demanding the notarizing of fraudulent bills for exorbitant sums of money (ranging from several tens of thousands of dollars, to billions—even trillions of dollars), or by directly challenging the legitimacy of judges in their own courtrooms. In a video posted to YouTube in 2010 by “Keith of the Thompson Clan,” a Sovereign Citizen in Manitoba, a man is seen directly challenging the judge's right to render a decision about court summons issued for illegally parking a vehicle on a lawn. The video clip has been viewed more than 500,000 times (LoudStudios, 2010). Such videos are by no means rare; a search of YouTube for the term “Sovereign Citizen” reveals hundreds—if not thousands—of channels and videos related to the subject. While many of these are discussions—and mockery—of SovCit beliefs, many more are produced by movement adherents discussing their strategies, struggles, and beliefs (MaKaElectric, 2012).

### Managing Status-Anxiety and Feelings of Inferiority

Unlike many other extremist groups on the political Right, Sovereign Citizens tend not to operate as parts of a larger social network, they instead act as individual agents sharing a loose collection of similar beliefs (Castells et al., 1996). This means that in practical terms, each act of resistance taken in opposition to the state or state representatives is done alone, or as part of a small, *ad-hoc* group with little long-term permanence, as was the case in the winter of 2015 when a small group of Sovereign Citizens and patriots led by Ammon Bundy occupied a bird sanctuary in rural Oregon (Zaitz, 2016). By far the most common example of Sovereign Citizen activism is found in actions taken by single SovCits or at most a pair of SovCits working in cooperation against a commonly-held opponent (Lenz and Potok, 2015). The radically individualist nature of Sovereign Citizenship would make larger, long-term projects more difficult as many SovCits have a strong distrust of any form of hierarchy or authority (Smith, 1997). This means that any act of deviance or resistance becomes an intensely personal one, and subsequently any triumph—or perceived triumph—need not be shared.

The importance of individual accomplishment and empowerment is centrally important to the process of counter-memorializing struggle against the state, for two inter-related reasons. The first is that in the immediate aftermath of the 2008 crisis, the economic shocks that rippled throughout the American economy created a sense of desperation among those most threatened by the crisis—people engaged in precarious labor and who also tend to be the target audience of people engaged in selling the strategies of the Sovereign Citizen movement (Southern Poverty Law Center, 2013). The economic crisis also magnified feelings of inferiority among some of those people who would later become SovCits; as states become increasingly neoliberal, feelings of inferiority become more individualized, resulting in a more isolated and fractured sense of social position (Neckel, 1996). Through this individualization of inferiority, individuals lose sight of larger patterns of social inequality, and come to view their position in society as a result of personal failings or personal inability to challenge the power of the state (Neckel, 1996). To a person faced with these challenges, the promise of personal sovereignty and the ability to choose to not be bound by the rules of the state is an enticing offer, not only because such a radical notion carries with it the promise of economic liberation, but also because the radical, Sovereign Citizen is an empowered, superior one. These beliefs are not restricted to Sovereign Citizen ideologies. Indeed, research into the rapid growth of the alt-right has shown how status anxiety has fueled not only radical individualism, but xenophobic, racist, Islamophobic, anti-Semitic, and transphobic ideologies across the political far Right (Hodge and Hallgrimsdottir, 2019).

The lure of these beliefs can be powerful: The state's control over a person's property is illegitimate; through proper recitation of the correct “truth language” phrases, any state court or legal authority can be made powerless. Through the correct application of another set of “truth language” invocations, a person can free themselves from personal debt, and gain access to the wealth that has been generated over the course of their entire lives through control over their corporate “straw man.” They are no longer bound by the arbitrary rules of the state, they are no longer bound by any legal authority at all; since all legal arrangements are voluntary, all it takes for a citizen to become sovereign is to say “no,” and they are free.

## Summary

While the Sovereign Citizen movement has ideological and philosophical roots stretching pack to anti-tax movements in the 1970s, the most recent wave of activity has emerged largely in response to the economic crisis and collapse of 2008. Conspiratorial in the extreme, individuals who adhere to Sovereign Citizen beliefs reject the authority of governments at almost every level above the county and even then, view authority or law enforcement personnel with great distrust. The primary objective of a Sovereign Citizen is to free themselves from the “illegitimate” authority of state and federal levels of government, and to gain control of a fictional pool of wealth they believe to be held in their name by their national government. Sovereign Citizens seek to gain access to this wealth using “truth language”; non-sensical pseudo-legal terms and phrases which, when recited in the correct order to the correct person will force state agents to cede their authority to the Sovereign Citizen.

To arrive at this worldview, Sovereign Citizens engage in a radical redefinition of citizenship rooted in the construction of counter-memories that oppose the traditional, hegemonic narrative of American or Canadian society. This counter-memory is then enacted using counter-memorializing—engaging in patterns of behavior or performance designed to signify the Sovereign Citizen's withdrawal of their consent to be governed by state or federal law. These counter-memorials include the self-issuing of fake passports, driver's licenses, or vehicle registrations, placing false liens against public properties or the private property of public officials, and directly challenging representatives of state or federal authority in face-to-face interactions like traffic stops, court appointments, or criminal trials. Through these actions, Sovereign Citizens signify their rejection of the rational-legal authority of the state and signal their adoption of a radically individualistic notion of personal sovereignty.

In the wake of the economic devastation of the 2008 global financial collapse, the Sovereign Citizen movement found new life, gaining a significant new following and claiming—by conservative estimates—over 100,000 active adherents in the United States, and roughly 30,000 adherents in Canada, with some sources placing the numbers of American SovCits at more than 300,000. The loss of security, coupled with heightened economic vulnerability and a perceived loss of control over financial matters led many to the movement, which promised to return control over their land, wealth, and persons at a time when many of them saw losses on these fronts. It was through the radical redefinition of citizenship promised by the movement's ideology that adherents came to believe that they could regain complete power and autonomy over their lives; every act of defiance or resistance became one more sign of a SovCit's regained power and self-worth. The 2008 global financial crisis was the catalyst for the movement's re-emergence and swift rise among members of the political Far Right, and the movement remains a powerful study in the performance of contested citizenship.

## Data Availability Statement

The datasets generated for this study will not be made publicly available the research is qualitative in nature; there are no datasets.

## Author Contributions

The author confirms being the sole contributor of this work and has approved it for publication.

### Conflict of Interest

The author declares that the research was conducted in the absence of any commercial or financial relationships that could be construed as a potential conflict of interest.

## Footnotes

^1^Legal filing submitted by former Ku Klux Klan leader and Sovereign Citizen (Barefoot, 2015).

^2^For example, consider this truth-language declaration from Sovereign Citizen and “plenipotentiary-judge” and creator of truth-language David-Wynn: Miller: “FOR THE NAME: “UNITED-STATES”, IN A COURT-ROOM-DOCUMENT IS WITH THE NAME-MEANING-CLAIM OF THE TWO-OR-MORE-CONTRACT-VESSEL-PERSONS IN THE DOCUMENT-CONTRACT-CORPORATION-VESSEL(D.-C.-C.-V.) AS THE TWO OR MORE-PERSONS WITHIN A CONTRACT-CLOSURE-PAPER-COURT-AREA. [HAS NOTHING TO DO WITH THE 50-USA-STATES]FOR ALL FOREIGN-COURTS OF A FOREIGN-GLOBAL-AREA WITH THE 7.6-BILLION-FOREIGN-PARSE-SYNTAX-GRAMMAR-PEOPLE ARE WITH THE CLAIM AS THE SINGLE-VESSEL IN A DRYDOCK-BUILDING WITH A CONTRACT AS THE COURT'S-“BILLS OF THE LAIDING” WITHIN THE LOCAL-PORT-AUTHORITIES-C.-S.-S.-C.-P.-S.-G.-TERRITORY.” (dwmlc.com 2016) A number of Sovereign Citizen books, blogs, and pamphlets state that part of the reason why national laws are of no force or effect on sovereign persons is because the laws are a twisted form of “admiralty law” or “law of the seas” with only govern the interactions of naval vessels (Southern Poverty Law Center, 2013).

^3^In activist or marginalized communities, counter-memorialization is in important method of object-ifying counter-memory. Examples of this include the creation of movement “days of remembrance” or the creation of statues or pieces of artwork that commemorate events the dominant culture has ignored or erased.
